# Amyloid β synaptotoxicity is Wnt-PCP dependent and blocked by fasudil

**DOI:** 10.1016/j.jalz.2017.09.008

**Published:** 2018-03

**Authors:** Katherine J. Sellers, Christina Elliott, Joshua Jackson, Anshua Ghosh, Elena Ribe, Ana I. Rojo, Heledd H. Jarosz-Griffiths, Iain A. Watson, Weiming Xia, Mikhail Semenov, Peter Morin, Nigel M. Hooper, Rod Porter, Jane Preston, Raya Al-Shawi, George Baillie, Simon Lovestone, Antonio Cuadrado, Michael Harte, Paul Simons, Deepak P. Srivastava, Richard Killick

**Affiliations:** a King's College London, Maurice Wohl Clinical Neuroscience Institute, London, UK; b The University of Manchester, Faculty of Biology, Medicine and Health, Division of Pharmacy and Optometry, Manchester, UK; c University of Oxford, Department of Psychiatry, Warneford Hospital, Oxford, UK; d Centro de Investigación Biomédica en Red sobre Enfermedades Neurodegenerativas (CIBERNED). Instituto de Investigación Sanitaria La Paz (IdiPaz), Autonomous University of Madrid, Madrid, Spain; e The University of Manchester, Faculty of Biology, Medicine and Health, Division of Neuroscience and Experimental Psychology, Manchester, UK; f Boston University School of Medicine, New England Geriatric Research Education and Clinical Center, Boston, USA; g Rod Porter, Rod Porter Consultancy, Baldock, England, UK; h King's College London, Institute of Pharmaceutical Science, Franklin-Wilkins Building, London, UK; i University College London, Centre for Amyloidosis and Acute Phase Proteins, Royal Free Campus, London, UK; j University of Glasgow, Institute of Cardiovascular and Medical Science, Glasgow, Scotland

**Keywords:** Dickkopf-1, Amyloid, Synapse, Synaptotoxicity, Wnt, Planar cell polarity, ROCK, DAAM1, Fasudil, Alzheimer's disease

## Abstract

**Introduction:**

Synapse loss is the structural correlate of the cognitive decline indicative of dementia. In the brains of Alzheimer's disease sufferers, amyloid β (Aβ) peptides aggregate to form senile plaques but as soluble peptides are toxic to synapses. We previously demonstrated that Aβ induces Dickkopf-1 (Dkk1), which in turn activates the Wnt–planar cell polarity (Wnt-PCP) pathway to drive tau pathology and neuronal death.

**Methods:**

We compared the effects of Aβ and of Dkk1 on synapse morphology and memory impairment while inhibiting or silencing key elements of the Wnt-PCP pathway.

**Results:**

We demonstrate that Aβ synaptotoxicity is also Dkk1 and Wnt-PCP dependent, mediated by the arm of Wnt-PCP regulating actin cytoskeletal dynamics via Daam1, RhoA and ROCK, and can be blocked by the drug fasudil.

**Discussion:**

Our data add to the importance of aberrant Wnt signaling in Alzheimer's disease neuropathology and indicate that fasudil could be repurposed as a treatment for the disease.

## Highlights

• Aβ synaptotoxicity is Dickkopf-1 and Wnt-PCP dependent.
• The Wnt-PCP pathway drives Aβ-driven synapse loss via RhoA and ROCK.
• ROCK inhibitor fasudil blocks Aβ-driven synapse loss and cognitive impairment.
• Fasudil should be assessed for repurposing for Alzheimer's disease.

## 1 Introduction

Amyloid β (Aβ) has long been associated with Alzheimer's disease (AD) through a propensity to form insoluble deposits, senile plaques, a hallmark of the AD brain. Overwhelming genetic and experimental evidence indicates that Aβ and its parent molecule, the Aβ precursor protein (APP), are key players in the neuropathogenic processes driving AD. Aβ readily self-associates to form a range of soluble oligomers and insoluble fibers, and the current consensus view holds that it is the small soluble oligomeric forms of Aβ rather than the plaques themselves that are the neurotoxic species [1], [2], [3]. We have previously found that Aβ-driven increases in tau phosphorylation (a second hallmark of the disease) and neuronal death are dependent on activation of a branch of Wingless/Wnt signaling known as the Wnt–planar cell polarity (Wnt-PCP) pathway, specifically the arm of Wnt-PCP acting through Jun N-terminal kinase (JNK) and its target, c-Jun, to regulate gene transcription [4] (please see glossary for definitions of these and other terms that occur below). We have shown that Aβ activates Wnt-PCP through the ability of Aβ to induce Dickkopf-1 (Dkk1). Dkk1 then blocks the binding interaction between LRP6 and frizzled, preventing canonical Wnt-β-catenin activity and concomitantly activating Wnt-PCP signaling [5], [6]. Furthermore, our data indicate that Dkk1 and Wnt-PCP not only shape the transcriptomic profile of the AD brain but also the activity of pathways within the brain most closely associated with the AD process [4], [7]. The top four most significant of these pathways are the adherens junction, Wnt signaling, TGF-β signaling, and long-term potentiation, all of which are intimately involved in synaptic plasticity [8], [9], [10].

Aβ synaptotoxicity is thought to be a very early event in the disease process, central to disease etiology and possibly the driver of many of the other neurotoxic properties attributed to Aβ [2], [11], [12]. Indeed, the degree of cognitive impairment in AD correlates more closely with synapse number than with amyloid load or extent of tau pathology [2], [13], [14]. However, although widely studied, the underlying mechanisms of Aβ synaptotoxicity have yet to be fully determined [2], [15].

In addition to influencing transcription via JNK/c-Jun, the Wnt-PCP pathway also regulates cytoskeletal dynamics through RhoA and ROCK, two key regulators of synapse formation [16], [17], both shown to be responsive to Aβ [18]. Given the synaptic effects of Aβ have been reported to be Dkk1 dependent [19] and as Dkk1 activates Wnt-PCP [4], we investigated the possibility that Aβ may exert its synaptotoxicity by activating the Wnt-PCP-RhoA/ROCK pathway via Dkk1 induction. We present evidence that this is indeed the case. Furthermore, we demonstrated the therapeutic potential of the ROCK inhibitor drug fasudil as a strategy to ameliorate both the synaptic and cognitive effects of Aβ.

## 2 Methods

### 2.1 Dkk1 measures

Rat Dkk1 mRNA expression was performed by *quantitative reverse transcription-polymerase chain reaction* and protein levels determined using a DuoSet ELISA kit (DY1906; R&D Systems), both as previously described [4].

### 2.2 Neuronal culture and transfections

Primary cortical neuronal cultures were prepared from Sprague-Dawley rat E18 embryos, as described previously [20]. Cells were seeded onto coverslips coated with poly-d-lysine 10 μg/mL (Sigma) at a density of 3 × 10^5^/well equating to 857/mm^2^. Cells were cultured in feeding media: neurobasal medium (21103049) supplemented with 2% B27 (17504044), 0.5 mM glutamine (25030024), and 1% penicillin/streptomycin (15070063) (all reagents from Life technologies, UK). After 4 days *in vitro* (d.i.v.), 200 μM of d,l-amino-phosphonovalerate (d,l-APV, ab120004; Abcam) was added to media to maintain neuronal health over long-term culture and to reduce cell death due to excitotoxicity [20]. Fifty percent media changes were performed twice weekly until desired time in culture was reached (23 d.i.v.). Cells were then transfected with an expression construct encoding enhanced green fluorescent protein (eGFP) driven by the synapsin 1 promoter using Lipofectamine 2000 resulting in 5%–10% transfection efficacy after 48 hours [20], [21]. The exogenous expression of eGFP is to allow the imaging of neuritic processes including dendritic spines, in those cells taking up the construct without the need for further labeling.

### 2.3 Pharmacological treatments of neuronal cultures

All pharmacological treatments were performed in artificial cerebral spinal fluid (aCSF): 125 mM NaCl, 2.5 mM KCl, 26.2 mM NaHCO_3,_ 1 mM NaH_2_PO_4_, 11 mM glucose, 5 mM HEPES, 2.5 mM CaCl_2,_ 1.25 mM MgCl_2_, and 0.2 mM APV. Neuronal cultures were pretreated with inhibitor compounds for 30 minutes before application of Dkk1 recombinant protein, Aβ_1–42_ oligomers, or fibrillar Aβ_25–35_. All compounds were dissolved in water or DMSO at a concentration of 10 or 1 mM, serially diluted to a 10× working concentration in aCSF, and applied directly to neuronal cultures. Final concentration of solvent was <0.01%, as also used in vehicle control. Treatments were allowed to proceed for indicated times before being fixed for immunocytochemistry.

### 2.4 Immunocytochemistry

Neurons were washed in phosphate-buffered saline (PBS) and then fixed in either 4% formaldehyde/4% sucrose in PBS for 10 minutes at room temperature followed by incubation in methanol prechilled to −20°C for 10 minutes at 4°C or in methanol (−20°C) only for 20 minutes at 4°C. Fixed neurons were then permeabilized and blocked simultaneously (2% Non-Immune Goat Serum; Sigma, and 0.2% Triton X-100) before incubation in primary antibodies overnight and subsequent incubation with secondary antibodies the next day [20]. In the green/purple color scheme, colocalization is indicated by white overlap.

### 2.5 Antibodies used

Green fluorescent protein, chicken polyclonal (ab13972; Abcam); PSD-95, mouse monoclonal (clone K28/43, 73-028; NeuroMab); PSD-95, rabbit polyclonal (2507; Cell Signaling Technology); Bassoon, mouse monoclonal (ab82958; Abcam); GluA1, rabbit polyclonal (ABN241; Millipore) are the antibodies used in this study.

### 2.6 Spine morphology and immunofluorescence

Images were acquired with the Leica SP-5 confocal microscope using a 63× oil-immersion objective (numerical aperture = 1.4; Leica) as z-series. Two-dimensional maximum projection reconstructions of images were generated, and morphometric analysis (spine number, area and breadth) was performed using MetaMorph software (Universal Imaging Corporation, West Chester, PA, USA) [20]. Morphometric analysis was performed on spines from at least two dendrites (secondary or tertiary branches), totaling 100 μm in length, per neuron. For each condition, 9–12 neurons from at least three separate experiments (each performed in duplicate) were used. Experiments were carried out blind to condition. Linear density and total gray value of each synaptic protein cluster were measured automatically using MetaMorph [20]. Cultures undergoing direct comparison were stained simultaneously and imaged with the same acquisition parameters.

### 2.7 Pharmacodynamics

Fasudil and hydroxyfasudil were administered separately at 10, 30, and 100 mg/kg, by intraperitoneal (i.p.) injection, to young adult male CD1 mice. Animals were sacrificed 20 minutes after dosing, and terminal plasma and brain samples were taken. Proteins were extracted using acetonitrile precipitation, and fasudil/hydroxyfasudil levels were measured by ultra high performance liquid chromatography - time of flight (UHPLC-TOF) mass spectrometry using electrospray ionization. CNS availability of fasudil was measured using an *in situ* rat-brain perfusion technique, as described previously [22]. Briefly, CNS circulation is isolated from the periphery through cannulation of the carotid arteries and ligation of jugular veins. Blood is replaced with plasma substitute containing fasudil and a vascular marker (e.g., sucrose) for up to 30 minutes. Whole brain homogenates are put through dextran centrifugation to remove brain capillaries, which may bind/trap drug. Fasudil and its metabolite (hydroxyfasudil) are measured by high-performance liquid chromatography in postcapillary brain parenchyma and in CSF sampled from the cisterna magna (25–50 μL sample collected by glass pipette before termination of perfusion).

### 2.8 Behavioral testing

#### 2.8.1 Animals

Female Lister-hooded rats (weighing ∼215 ± 20 g at the start of experimentation; Charles River, UK) were housed in groups of five in individually ventilated two-story home cages, in a 12-hour light cycle (illuminated 07:00 to 19:00 hours) with controlled temperature (21 ± 2°C) and humidity (55 ± 5%). Water and food (Special Diet Services, UK) were given ad libitum. All experiments were undertaken during the illuminated period and conducted in accordance with UK Animals (Scientific Procedures) 1986 Act and the University of Manchester ethical guidelines.

#### 2.8.2 Aβ_1–42_ oligomer preparation

Biotin-Aβ_1–42_ (ANA24640) was purchased from AnaSpec, USA, disaggregated in hexafluoroisopropanol for 1 hour, aliquoted, and hexafluoroisopropanol was removed by evaporation under N_2_; and the monomeric peptide was solubilized in DMSO at 1 mM, diluted to 100 μM in Ham's F12, and allowed to oligomerize at room temperature for 16 hours.

#### 2.8.3 Surgical procedure

Rats were anesthetized using 4% isoflurane in O_2_ in an induction chamber, mounted in a stereotaxic frame, and anesthesia was maintained with 2%–3% isoflurane. Then, 10 μL of 100 μM Aβ_1–42_ oligomers (AβO) was injected into the left lateral ventricle using Bregman coordinates—H-0.8; Tr-1.5; V-4.5, at a flow rate of 2.5 μL/min (total administered = 10 μmol). Surgery date was defined as day 0. Rats were treated with i.p. injections of vehicle or fasudil at 10 mg/kg twice daily from day 1 to day 6. The novel object recognition (NOR) test was performed on day 7, as previously described [23]. In brief, rats were placed in a 52- × 52- × 51-cm polyvinyl chloride arena for 3 minutes with two identical objects. Animals were taken out of the box for an intertrial interval of 1 minute then placed back in the same box for further 3 minutes with an identical copy of the previous object and a novel object. Both sessions were digitally recorded, and the time spent in exploring each object was scored. The discrimination index was calculated as (novel − familiar)/(novel + familiar).

### 2.9 Statistical analyses

Statistical analyses were performed in GraphPad or SPSS. Differences in quantitative immunofluorescence, dendritic spine number, and morphology were identified by Student's unpaired t-tests. For comparisons between multiple conditions, the main effects and simple effects were probed by 1- or 2-way analyses of variance with Tukey's correction for multiple comparisons.

## 3 Results

### 3.1 Aβ drives Dkk1 production

**Fig. 1:**
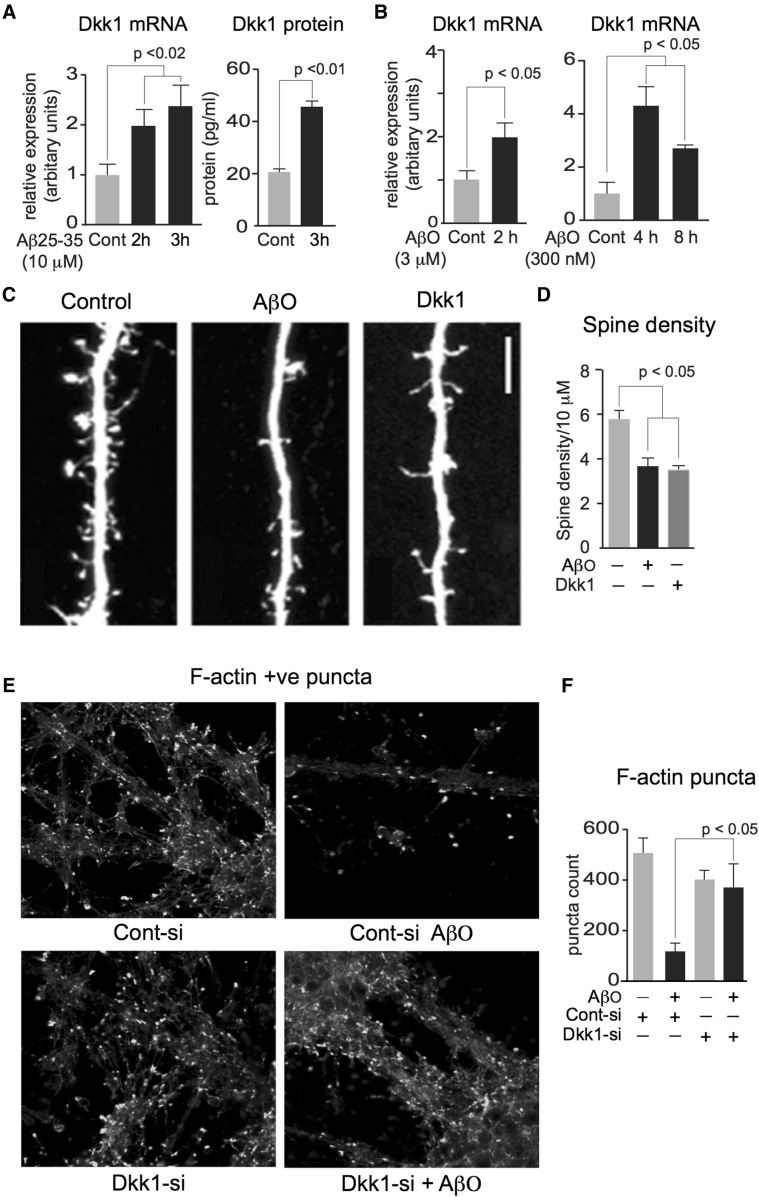
Aβ synaptotoxicity is Dkk1 dependent. (A) Rat primary cortical neuronal cultures (14 d.i.v.) were treated with 10 μM Aβ_25–35_ for 2 and 3 hours, cells were harvested for RNA extraction, and media were collected for protein analysis. cDNA was generated and qRT-PCR performed to determine rat Dkk1 mRNA levels, left. Secreted Dkk1 protein levels in media were measured by ELISA, right. (B) Similar cultures were treated at 3 μM and 300 nM with AβO preparation for the times indicated and harvested, and Dkk1 mRNA levels were determined as mentioned previously. (C and D) Similar cultures were transfected with eGFP at 24 d.i.v., 48 hours later treated for 4 hours with 2 μM AβO, or for 3 hours with 400 ng/mL Dkk1, fixed, imaged by confocal microscopy, and dendritic spine density and morphology assessed. Both treatments resulted in a significant reduction in dendritic spine linear density, quantified in (D), scale bar = 5 μM. Dendritic spine linear density/10 μm: control, 5.8 ± 0.41; AβO, 3.7 ± 0.34; Dkk1, 3.5 ± 0.31; *P* < .001 for all treatments. (E and F) Similar cultures were treated overnight with Dkk1-siRNA duplex, or a scrambled version as control, each linked to the Pen-1 peptide. Next day, cells were treated with 2 μM AβO for 4 hours, fixed, and fluorescently labeled with phalloidin-488, imaged (E), and F-actin–labeled puncta quantified (F), scale bar = 50 μM. In all the aforementioned, significance was determined by ANOVA and Tukey's post hoc t-test. Error bars indicate standard error of the mean. Abbreviations: AβO, amyloid β (1–42) oligomers; ANOVA, analysis of variance; d.i.v., days in vitro; eGFP, enhanced green fluorescent protein; Dkk1, Dickkopf-1.

Aβ drives Dkk1 expression [4], [19], [24], and Dkk1 protein levels are raised in the brains of Aβ/APP-based mouse models of AD pathology [25]. We extend these observations by showing that increases in both Dkk1 mRNA and protein are readily detectable in cultured rodent neurons following treatment with the active portion of Aβ, Aβ_25–35_, and by soluble AβO within 2–3 hours and within 4 hours by AβO at nanomolar concentrations (Fig. 1A and 1B).

### 3.2 Synaptic effects of Aβ and Dkk1 are similar

To compare the synaptic effects of Aβ and Dkk1 protein, rat cortical neurons (24 d.i.v.) were transfected with eGFP and 48 hours later treated with either 2 μM AβO for 4 hours or with 400 ng/mL recombinant Dkk1 proteins for 3 hours. An additional 1 hour was given for Aβ treatments to allow time for endogenous Dkk1 expression, thereby rendering the two treatments more comparable. AβO and Dkk1 had potent, significant, and very similar effects on dendritic spine linear density (Fig. 1C and 1D). After exposure to either AβO or Dkk1, a small number of immature filopodia-like dendritic spines appear to be spared indicating that Aβ-driven spine loss is selectively targeting dendritic spines with established postsynaptic densities, an observation we are now investigating in more detail.

### 3.3 Aβ synaptotoxicity is Dkk1 dependent

Antibody neutralization of Dkk1 blocks Aβ-induced synapse loss [19]. To extend this, we knocked down Dkk1 expression in primary cortical neuronal cultures using a previously validated penetrating peptide-coupled siRNA duplex targeting rat Dkk1 with a scrambled form as control [4]. Assessing synapse number by phalloidin-488 labeling of F-actin puncta in the presence of the control siRNA, 2 μM AβO caused a substantial and significant reduction in synapse number for more than 4 hours. This was significantly blocked in neurons treated with the Dkk1-siRNA (Fig. 1E and 1F), confirming that Dkk1 is required for Aβ synaptotoxicity to occur.

### 3.4 Dkk1 synaptotoxicity is Daam1 dependent

**Fig. 2:**
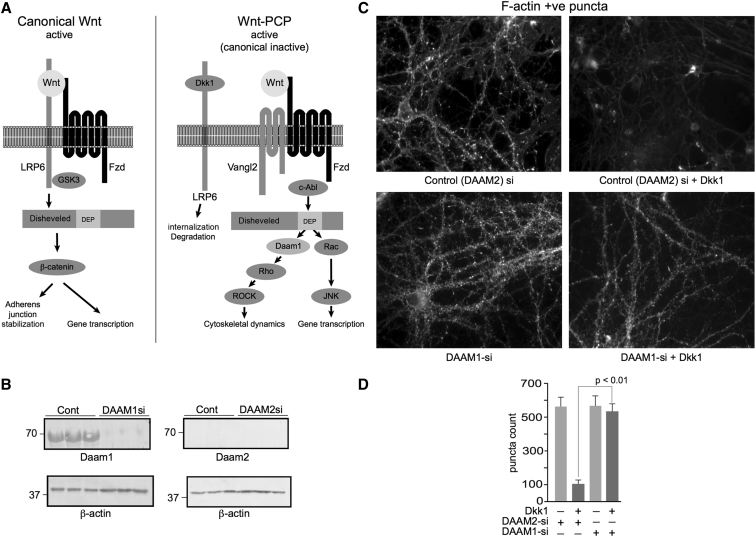
Dkk1 synaptotoxicity is Daam1 dependent. (A) Schematic of the Wnt-PCP pathway, showing the two arms branching below disheveled, acting via Daam/Rho/ROCK to regulate cytoskeletal dynamics and JNK/c-Jun primarily to regulate gene transcription. (B) Primary cortical neuronal cultures were treated with DAAM1 or DAAM2 Pen-1–coupled siRNA duplexes for 48 hours, harvested, and analyzed by Western blotting for Daam1 and Daam2. Daam1 was detectable in untreated cells, whereas Daam2 was not. Daam1-si potently reduced Daam1 protein expression levels. (C and D) Cultures were treated overnight with DAAM1- or DAAM2-siRNA duplexes. Next day, cells were treated with 400 ng/mL recombinant Dkk1 protein for 3 hours, fixed, and fluorescently labeled with phalloidin-488, imaged (C), scale bar = 50 μM, and F-actin–labeled puncta quantified (D). Significance determined by ANOVA and Tukey's post hoc t-test. Error bars indicate standard deviation. Abbreviations: Aβ, amyloid β; ANOVA, analysis of variance; c-Abl1, c-Abl oncogene 1, nonreceptor tyrosine kinase (ABL1); d.i.v., days in vitro; DAAM1, disheveled associated activator of morphogenesis 1; Dkk1, Dickkopf-1; Dvl, disheveled; EGR1, early growth response 1; Fzd, frizzled; GSK3-α/β, glycogen synthase kinase-α/β; JNK1, c-Jun N-terminal kinase (MAPK8); KLF10, Krüppel-like factor 10; LRP6, low-density lipoprotein receptor–related protein 6; MKK4/7, mitogen-activated protein kinase 4/7 (MAP2K4 and MAP2K7); NAB2, NGFI-A binding protein 2; PCP, planar cell polarity; RhoA, Ras homolog family member A; ROCK, Rho-associated coiled-coil containing protein kinase; Vangl2, Van Gogh–like protein 2.

Activation of the Wnt-PCP/RhoA/ROCK pathway requires an interaction between disheveled and Daam1 or Daam2 (Fig. 2A). The only known role of Daam1 and Daam2 is as elements of the Wnt-PCP pathway [26], [27], [28]. To determine if Dkk1-driven synapse loss is truly Wnt-PCP dependent, we individually knocked down either DAAM1 or DAAM2 in rat primary cortical cultures using penetrating siRNA duplexes before Dkk1 treatment. Western blotting of treated cultures demonstrates that only Daam1 is expressed at detectable levels in these cells, in agreement with previous reports that Daam1 is the predominant isoform in neurons [29], and that DAAM1-siRNA treatment potently reduced Daam1 protein expression after 48 hours (Fig. 2B). Given the lack of detectable Daam2 expression, we used a DAAM2 siRNA as an appropriate control. Cortical neuronal cultures (24 d.i.v.) were then treated for 3 hours with recombinant Dkk1 protein (400 ng/mL) or vehicle (PBS) after DAAM1 or DAAM2 silencing. Cells were then fixed, and synapse number was assessed by counting phalloidin-labeled F-actin puncta. Silencing of DAAM1 or DAAM2 did not affect synapse homeostasis, as puncta counts were equivalent to untreated controls. However, Dkk1-driven synapse retraction, as demonstrated by a substantial reduction in puncta, was only observed after silencing DAAM2 and was ameliorated by siRNA knockdown of DAAM1 (Fig. 2C and 2D). These data indicate that Dkk1-mediated synapse loss occurs through the activation of the Wnt-PCP pathway in a Daam1-dependent manner and demonstrates that this mechanism is pivotal for synapse loss but not synapse maintenance, as silencing DAAM1 alone had no deleterious effect on synapse stability.

### 3.5 Aβ-driven, Dkk1-dependent spine loss is mediated by RhoA/ROCK

**Fig. 3:**
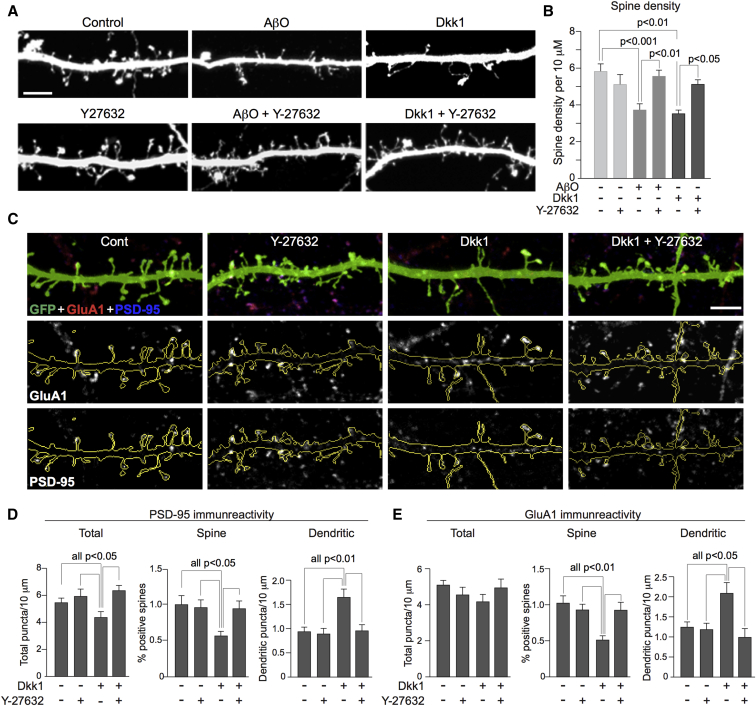
Aβ and Dkk1 synaptic effects are ROCK dependent. (A and B) Rat primary cortical neurons were transfected with eGFP at 24 d.i.v. and 48 hours later treated with Y-27632 or vehicle, and 15 minutes later with 2 μM AβO for 4 hours or 400 ng/mL Dkk1 for 3 hours, fixed, and imaged by confocal microscopy for the examination of spine morphology. AβO and Dkk1 caused a significant reduction in dendritic spine linear density. Y2763 alone had no significant effect on spine density, but in combination with AβO and Dkk1, it blocked the effect of both (scale bar = 5 μM). (C, D, and E) Rat primary 26-d.i.v. neurons expressing eGFP were treated with Y-27632 and Dkk1, all as mentioned previously (3A). (D) Concurrent with a loss of spine density, Dkk1 caused a significant reduction in total PSD-95 puncta (Total PSD-95 density/10 μm: control, 5.5 ± 0.34; control + Y-27632, 6.2 ± 0.55; Dkk1, 4.3 ± 0.39; Dkk1 + Y-27632, 6.6 ± 0.43). Interestingly, the number of spines containing PSD-95 was also reduced, with a concurrent increase in the density of dendritic PSD-95, following treatment with Dkk1. This effect was blocked by Y-27632 (% spines containing PSD-95: control, 79.8 ± 2.4; control + Y-27632, 78.2 ± 2.7; Dkk1, 59.1 ± 3.7; Dkk1 + Y-27632, 78.9 ± 2.1). Dendritic PSD-95 puncta/10 μm: control, 0.93 ± 0.11; control + Y-27632, 0.89 ± 0.11; Dkk1, 1.67 ± 0.17; Dkk1 + Y-27632, 0.95 ± 0.15. (E) Dkk1 did not significantly affect the total level of GluA1-immunoreactive puncta but did reduce the number of spines positive for GluA1 and increased levels of GluA1 in dendrites, which was again blocked by inhibition of ROCK. (1) GluA1 linear density/10 μm: control, 5.0 ± 0.34; control + Y-27632, 4.6 ± 0.40; Dkk1, 4.2 ± 0.40; Dkk1 + Y-27632, 4.9 ± 0.44; *P* = .4574; (2) % spines containing GluA1: control, 67.8 ± 4.3; control + Y-27632, 68.4 ± 3.7; Dkk1, 47.9 ± 4.4; Dkk1 + Y-27632, 65.6 ± 3.1; and (3) Dendritic GluA1 puncta/10 μm: control, 1.25 ± 0.13; control + Y-27632, 1.20 ± 0.15; Dkk1, 2.09 ± 0.26; Dkk1 + Y-27632, 0.99 ± 0.22. In all bar graphs, error bars indicate standard error of the mean. Abbreviations: AβO, amyloid β (1–42) oligomers; Dkk1, Dickkopf-1; eGFP, enhanced green fluorescent protein; ROCK, Rho-associated coiled-coil containing protein kinase.

Downstream of Daam1 Wnt-PCP regulates actin cytoskeletal dynamics through RhoA and ROCK [30], [31]. We therefore investigated whether pharmacological inhibition of the RhoA/ROCK pathway would inhibit both AβO- and Dkk1-driven spine losses. Cortical cultures were transfected with eGFP at 23 d.i.v. and 48 hours later pretreated with the well-characterized ROCK inhibitor, Y-27632, or with vehicle for 30 minutes, and then with 2 μM AβO for 4 hours or with 400 ng/mL Dkk1 recombinant protein for 3 hours. Assessment of dendritic spine linear density showed that Y-27632 blocked both AβO- and Dkk1-induced spine losses with similar potency, while Y-27632 alone had no effect on spine number (Fig. 3A and 3B). Together with the aforementioned (Fig. 1c–1g and Fig. 2c and 2d), these data provide strong evidence that Aβ synaptotoxicity is dependent on Dkk1-driven activation of a Wnt-PCP-Daam1-RhoA/ROCK pathway.

### 3.6 Dkk1 drives GluA1 and PSD-95 relocation

Acute Aβ exposure causes a reduction in synaptic transmission through the internalization of AMPA (α-amino-3-hydroxy-5-methyl-4-isoxazolepropionic acid) receptors [32], [33], while Dkk1 has been suggested to cause a removal of PSD-95 from synapses [19]. However, whether acute exposure to Dkk1 drives the removal of PSD-95 away from dendritic spines and the internalization of AMPA receptors is not known. To investigate this and determine whether effects on PSD-95 and GluA1-containing AMPA receptors could also be blocked by ROCK inhibition, 26-d.i.v. eGFP-expressing cortical neurons were pretreated with Y-27632 or vehicle and subsequently by Dkk1 for 3 hours. After fixation and immune-labeling for PSD-95 and GluA1, confocal imaging revealed that Dkk1 treatment causes a significant reduction in the total number of PSD-95–positive puncta with significantly fewer PSD-95–positive spines while concurrently increasing PSD-95–immunoreactive puncta within dendrites. These effects were blocked by Y-27632 (Fig. 3C and 3D). Dkk1 did not affect the total level of GluA1 puncta but did similarly reduce the number of GluA1-positive spines and increase GluA1 immunolabeling within the dendritic shaft, which was again blocked by Y-27632 (Fig. 3C and 3E). Dkk1 induces the removal of PSD-95 and GluA1 proteins from synapses and promotes their trafficking into dendrites, similar to that observed with Aβ. This also confirms that Dkk1-induced dendritic spine loss is concomitant with a loss of synapses via a RhoA/ROCK-dependent mechanism.

### 3.7 Aβ- and Dkk1-induced spine withdrawals are blocked by fasudil

**Fig. 4:**
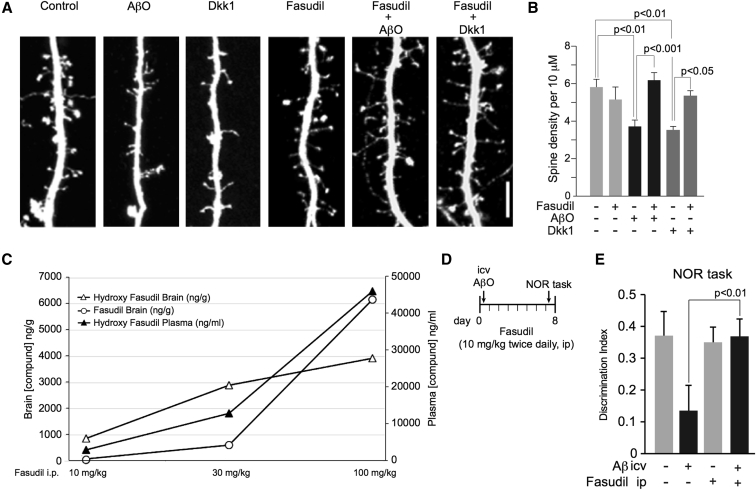
Fasudil is CNS penetrant and blocks Aβ synaptotoxicity and cognitive impairment. (A and B) Rat primary cortical neuronal cultures were transfected with eGFP at 26 d.i.v. and 48 hours later pretreated with 5 μM fasudil or vehicle for 15 minutes and subsequently treated with AβO or Dkk1 and imaged as mentioned in Fig. 3A, scale bar = 5 μM. AβO and Dkk1 caused a significant reduction in dendritic spine linear density, which fasudil fully and significantly blocked, as shown in (B) (dendritic spine linear density/10 μm: control, 5.8 ± 0.41; fasudil, 5.2 ± 0.64; AβO, 3.7 ± 0.34; AβO + fasudil, 6.2 ± 0.38; Dkk1, 3.5 ± 0.20; Dkk1 + fasudil, 5.4 ± 0.27). (B) Significance determined by ANOVA and Tukey's post hoc t-test. In (B), error bars indicate standard error of the mean. (C) Male CD1 mice were administered fasudil or hydroxyfasudil at 10, 30, and 100 mg/kg, ip, and brain and plasma were collected 20 minutes after injection. Fasudil and hydroxyfasudil were detected and measured by mass spectrometry. Plasma levels of fasudil were below the threshold of detection at all doses, and its data points were omitted from the graph. (D and E) 40 young adult female rats were administered 10 mg/kg fasudil, or vehicle, ip, twice daily for 7 days, and given either a single dose of AβO or vehicle, unilaterally, icv, on day 1. On day 7, all animals were presented with an NOR task, schematized in (D) Rats receiving vehicle and AβO showed profound deficit in this task, while the performance of rats receiving AβO and fasudil was not different to that of controls (E) Error bars in (E) indicate standard deviation. Abbreviations: AβO, amyloid β (1–42) oligomers; Dkk1, Dickkopf-1; NOR, novel object recognition; eGFP, enhanced green fluorescent protein; icv, intracerebroventricularly; ip, intraperitoneally.

To confirm the effects of Y-27632 (a hexane carboxamide) are via ROCK, we selected a second structurally dissimilar ROCK inhibitor, fasudil (an isoquinoline). Fasudil is one of the only two ROCK inhibitors approved for clinical use [34]. eGFP-expressing cortical cultures were pretreated with 5 μM fasudil or vehicle for 30 minutes and subsequently with either 2 μM AβO or 400 ng/mL Dkk1 recombinant protein, fixed and imaged by confocal microscopy. AβO and Dkk1 both significantly reduced spine number, and the effects of each were again blocked by fasudil (Fig. 4A and 4B), while fasudil treatment alone, like Y-27632, had little effect on spine density. These data support our contention that the synaptic effects of both Aβ and Dkk1 are dependent on the Wnt-PCP-RhoA/ROCK pathway and substantiate the synaptoprotective properties only very recently attributed to fasudil [35].

### 3.8 Fasudil rescues Aβ-driven cognitive deficits

Fasudil has clinical approval and protects against Aβ synaptotoxicity, which make it a promising candidate for repositioning for AD. To further assess its usefulness for this purpose, we examined its ability to protect against AβO-induced cognitive impairment *in vivo* using a novel acute rat model [36]. Despite the predicted low CNS penetrance of fasudil [37], both fasudil [38] and its active metabolite hydroxyfasudil [39] appear to be centrally active after peripheral administration. Given the paucity of data concerning brain availability of either compound and to inform on dosing for *in vivo* experimentation, we evaluated brain penetrance of fasudil and hydroxyfasudil. Each was administered at a range of doses by i.p. injection to CD1 mice, and levels of each were measured in brain and plasma by mass spectrometry. Plasma levels of fasudil were below detection threshold at this time point because of it being rapidly metabolized into hydroxyfasudil. However, these data demonstrate that fasudil and hydroxyfasudil are both brain penetrant (Fig. 4C) and that fasudil that enters the CNS persists there for some considerable time. Data obtained by the rat *in situ* brain perfusion technique also demonstrate that fasudil is brain penetrant with a plasma-brain ratio of 8.5% at a 10-mg/kg dose (Fig. 4C). This ratio is better than that of a number of compounds widely used to treat CNS disorders such as clozapine (1.1%), haloperidol (1.1%), and diazepam (3.6%) and is similar to that of donepezil, 12.6% [40], one of the few drugs currently licensed for AD.

Based on these data, 40 adult female rats of 250–300 g body weight were administered fasudil (10 mg/kg) or vehicle (saline), intraperitoneally twice daily for 7 days. Following the initial i.p. injection, all animals underwent surgery on day 1 to receive a single intracranial injection into the left lateral ventricle of either 10 nmol AβO or vehicle in a volume of 10 μL (at 2.5 μL/min) resulting in four groups (n = 10/group). On day 7, all 40 animals were presented with an NOR task, as previously described [41]. In rats receiving vehicle, the single dose of AβO produced a marked and highly significant impairment in the NOR task performance compared with controls. Fasudil alone had no effect on the NOR task performance but completely rescued performance deficits due to AβO (Fig. 4C). These data confirm that peripherally administered fasudil can block AβO-driven cognitive impairment. Given the supporting evidence we presented previously, we propose that fasudil is able to protect against Aβ-induced cognitive impairment through its ability to antagonize an Aβ-activated Dkk1-Wnt-PCP-Daam1-RhoA/ROCK-dependent pathway that drives dendritic spine withdrawal and synapse loss.

## 4 Discussion

Opposing roles for the canonical and noncanonical Wnt-signaling pathways in synapse homeostasis have been previously recognized, with canonical Wnt-promoting synapse formation and stabilization [42] and noncanonical Wnt-promoting synapse disassembly/pruning [43], [44]. Under normal physiological conditions, both pathways likely act in a highly regulated and coordinated manner to achieve appropriate levels of synaptic plasticity and normal cognitive functioning. Abnormal levels of Aβ result in cognitive impairment and memory deficits by disrupting these processes.

Our data demonstrate that Aβ-driven synapse withdrawal involves the Dkk1-dependent activation of the Wnt-PCP-RhoA/ROCK pathway. We show that at nanomolar levels, oligomeric forms of Aβ_1–42_ regarded to be the most synaptotoxic form of Aβ [45], [46], rapidly upregulate neuronal Dkk1 expression leading to dendritic spine retraction and altered localization of the postsynaptic proteins, PSD-95, and GluA1, and that these effects are dependent on Daam1 and ROCK.

It has been postulated that Dkk1 alters synapse stability predominantly through antagonism of the canonical Wnt-β-catenin pathway [19], which doubtlessly contributes to the process given the recognized role of canonical Wnt in synapse formation and stability [42], [47]. Our data significantly advance on this idea, demonstrating that Dkk1-mediated synapse loss involves the simultaneous and necessary activation of the Wnt-PCP-RhoA/ROCK pathway. This is in line with previous reports specifically pointing to a role of Wnt-PCP in synapse disassembly through the core PCP component Vangl2 [43], [44]. Taking this further, Aβ induction of Dkk1 likely exerts two simultaneous effects both detrimental to synaptic connectivity, a reduction in synaptic adherens junction stability due to a reduction in β-catenin levels by antagonizing the canonical Wnt pathway and concomitantly allowing activation of Wnt-PCP that acts on cytoskeletal dynamics to directly drive synapse withdrawal.

**Fig. 5:**
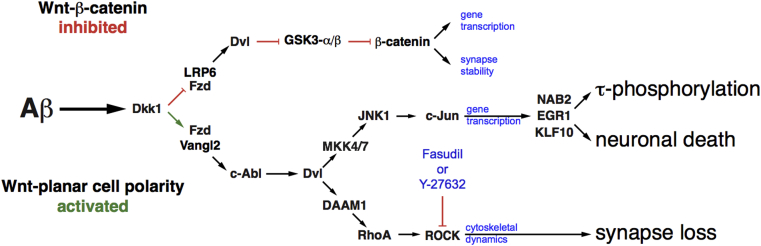
Schematic of Aβ-driven Wnt-PCP pathway activation. Aβ drives a rapid increase in Dkk1 expression. Concomitant with antagonism of canonical Wnt-β-catenin–signaling Dkk1 then drives the activation of the Wnt-PCP pathway by antagonizing the LRP6-Fzd interaction. We have previously shown that activity in the JNK/c-Jun arm of Wnt-PCP induces the expression of several identified genes required for Aβ-driven increases in tau phosphorylation and neuronal death to occur. Here, we demonstrate that activity of the Daam1/RhoA/ROCK arm is necessary for Aβ-driven synaptotoxicity and that this can be blocked by ROCK inhibitors Y-27632 or fasudil. Abbreviations: Aβ, amyloid β; c-Abl1, c-Abl oncogene 1, nonreceptor tyrosine kinase (ABL1); DAAM1, disheveled associated activator of morphogenesis 1; Dkk1, Dickkopf-1; Dvl, disheveled; EGR1, early growth response 1; Fzd, frizzled; GSK3-α/β, glycogen synthase kinase-α/β; JNK1, c-Jun N-terminal kinase (MAPK8); KLF10, Krüppel-like factor 10; LRP6, low-density lipoprotein receptor–related protein 6; MKK4/7, mitogen-activated protein kinase 4/7 (MAP2K4 and MAP2K7); NAB2, NGFI-A binding protein 2; PCP, planar cell polarity; RhoA, Ras homolog family member A; ROCK, Rho-associated coiled-coil containing protein kinase; Vangl2, Van Gogh–like protein 2.

We previously reported that Aβ, through Dkk1, aberrantly activates the JNK/c-Jun arm of Wnt-PCP, which then drives the expression of genes required for Aβ-induced neuronal death and increases in tau phosphorylation *in vitro* and *in vivo* [4]. Furthermore, we also presented evidence that the signaling pathways most associated with disease in the AD brain are shaped, if not driven, by Dkk1-Wnt-PCP activity [4]. We then now argue that the Aβ-driven Dkk1-dependent activation of Wnt-PCP underpins multiple of the key neuropathological characteristics of AD including, possibly the most fundamental of all, the loss of synaptic connectivity. This concept is depicted schematically in Fig. 5 in which we also indicate the dual effects of Aβ induction of Dkk1, it not only inhibiting the canonical Wnt pathway but concomitantly permitting activation of the Wnt-PCP pathway.

Although we hold that a dysregulation of Wnt signaling is likely to be central to AD pathology, work from other groups, strongly supported by genetic evidence, also clearly indicates an involvement of other systems and pathways. The main players that have emerged are immunity/inflammation and endocytosis/autophagy. In addition, the major tau kinase GSK3 also remains a key player in the disease, and although it occupies a central position in Wnt signaling, it is significant in many alternative pathways, particularly in insulin and p53 signaling that have both been strongly implicated in AD.

Given the above, the fact that the familial AD gene APP (the parent molecule of Aβ) has itself recently been shown to be a component of the Wnt-PCP co-receptor complex surely underpins the importance of this pathway in the disease process [48]. It also indicates that a better understanding of the both the physiological and the pathological roles of both Aβ and APP in this pathway will shed further light on the mechanism and improve our ability to therapeutically intervene in a effective manner to slow it down or even prevent it.

Here, not only do we shed new light on these mechanisms but also we identify fasudil, a drug that has been approved for clinical use in Japan and China since 1994 for cerebrovascular vasospasm, as a strong candidate for repositioning/repurposing for AD. We assessed the pharmacodynamics of fasudil and its active metabolite hydroxyfasudil, showing both have good brain penetrance. Owing to legal infringements within the pharmaceutical industry, fasudil has not received the Food and Drug Administration or European approval. However, in China, it has been used in a small clinical trial in AD patients in combination with a second vasodilator, nimodipine. In this study, fasudil was found to improve cognitive function compared with nimodipine alone [49].

A recent report has shown that ROCK inhibitor, Y-27632, can reverse Dkk1-induced synapse loss *in vivo* [47]. Thus, that fasudil is well tolerated in humans [50] the data we present here concerning its ability to protect against Aβ synaptotoxicity, to have good brain availability, and to protect against Aβ-induced cognitive impairment, warrants serious assessment of its utility as a much needed treatment for AD.

**Research in Context**

1. Systematic review: Several decades of medical research strongly indicate that synapse loss is an early and key event in Alzheimer's disease and that this is driven by soluble oligomeric forms of the amyloid β (Aβ) peptide. However, the molecular mechanisms underlying Aβ synaptotoxicity are not clear, nor has any medication been identified that can halt this.
2. Interpretation: We present strong evidence that Aβ-driven synapse loss is dependent on a branch of Wnt signaling known as the planar cell polarity pathway. In elucidating this mechanism, we found that synapses, and cognition in rats, are protected from the effects of Aβ by a drug in clinical use, fasudil.
3. Future directions: These findings will allow a yet more detailed understanding of the mechanisms controlling the synaptic effects of Aβ to be determined. Importantly, they indicate that fasudil, which is safe in humans and readily enters the brain, is a very promising candidate treatment for Alzheimer's disease.

## Glossary

Canonical Wnt: The branch of Wnt signaling dependent on β-catenin, a protein that has dual roles as an adhesion molecule at the cell membrane and as a regulator of gene transcription in the nucleus.
c-Jun: v-Jun Avian sarcoma virus 17 oncogene homolog, a transcription factor also known as activator protein 1 (AP1).
Daam: Disheveled associated activator of morphogenesis, a regulator of cell motility and adhesion acting on the actin cytoskeleton. In mammals, there are two homologous genes, DAAM1 and DAAM2.
Dkk1: Dickkopf-1 antagonizes canonical Wnt signaling by inhibiting LRP6 interaction with Wnt and promotes internalization of LRP5/6.
GSK3: Glycogen synthase kinase 3.
JNK: JUN N-terminal kinase, also known as mitogen-activated protein kinase 8.
LRP6: Low-density lipoprotein receptor–related protein 6, a necessary co-receptor for canonical Wnt.
Planar Cell Polarity (PCP): The planar orientation of cells within an epithelia sheet. An example of this in mammals is the orientation of auditory hair cells in the inner ear.
ROCK: Rho-associated coiled-coil containing protein kinase. Protein kinase involved in the regulation of actin cytoskeleton and cell polarity.
TGF-β: Transforming growth factor β, a multifunctional cytokine.
Vangl: Vang (Van Gogh, *Drosophila*)-like, a Wnt-PCP co-receptor component. In mammals, there are two homologous genes, VANGL1 and VANGL2.
Wingless: a locus in the fly, *Drosophila melanogaster*. Mutations that may result in a phenotype that includes the loss of wings. The Wingless gene (*wg*) encodes a secreted protein that binds the frizzled receptor.
Wnt: Wingless is known as Wnt in vertebrates, a contraction of Wingless and int1, which was coined when it was discovered that integration site 1 (int1) of the mouse mammary tumor virus (MMTV) was in a gene homologous to *wg*.
Wnt-PCP: The branch of Wnt signaling regulating planar cell polarity.
